# Visualization and Detection of Ciliary Beating Pattern and Frequency in the Upper Airway using Phase Resolved Doppler Optical Coherence Tomography

**DOI:** 10.1038/s41598-017-08968-x

**Published:** 2017-08-17

**Authors:** Joseph C. Jing, Jason J. Chen, Lidek Chou, Brian J. F. Wong, Zhongping Chen

**Affiliations:** 1 Beckman Laser Institute, University of California, Irvine, 1002 Health Sciences Road, Irvine, 92617 CA USA; 2 Department of Biomedical Engineering, University of California, Irvine, 3120 Natural Sciences II, Irvine, 92697-2715 CA USA; 3 Department of Otolaryngology—Head and Neck Surgery, University of California, Irvine, 101 The City Drive South, Orange, 92868 CA USA

## Abstract

Ciliary motion plays a critical role in the overall respiratory health of the upper airway. These cilia beat at a native frequency and in a synchronized pattern to continuously transport foreign particulate trapped in a layer of mucous out of the upper airway. Disruption of ciliary motion can lead to severe respiratory diseases and compromised respiratory function. Currently, the study of cilia requires expensive high speed cameras and high powered microscopes which is unsuitable for *in vivo* imaging and diagnosis. Doppler based optical coherence tomography has the potential to visualize the microscopic motion of cilia during their beating cycle. We demonstrate the development of a high-speed Doppler optical coherence tomography system that not only can rapidly determine the cilia beat frequency, but also simultaneously visualize the temporal cilia beating pattern which plays critical roles in cilia function.

## Introduction

Outside of serving as the entrance to the respiratory system, the upper airway has a critical role in the immune system. The majority of the upper airway is lined by a delicate respiratory epithelium that acts as the first line of defense from airborne pathogens. A layer of mucus is secreted along the surface that serves to both maintain epithelial moisture and trap foreign particulates. This mucus is continuously transported by the synchronized rhythmic beating of microscopic cilia towards the pharynx where it is either swallowed or expelled through the oral cavity. These cilia are tiny hair-like structures that line the surface of epithelial cells and are approximately 5–7 μm long, less than 1 μm in diameter^1, 2^, and beat at a frequency estimated to vary from 7 to 16 Hz^3–5^. Ciliary beat frequency (CBF) depends on temperature and humidity and changes with exposure to drugs and noxious stimuli. Abnormal ciliary function can lead to inadequate mucociliary clearance (MCC) which is associated with various respiratory diseases such as cystic fibrosis (CF), chronic obstruction pulmonary disease (COPD) and sinusitis. Disruption of cilia synchronicity also leads to poor MCC as seen in primary ciliary dyskinesia (PCD) which can result in chronic respiratory infections^3, 6^. Therefore, measurement of ciliary dynamics can serve as an important indicator of upper respiratory health.

The vast majority of CBF measurements in the upper airway are made using *in vitro* or *ex vivo* tissue samples. A cytology brush is commonly used to obtain cells from the nasal turbinate which are then either incubated or grown in a tissue culture. Brushing can be noxious for the patient and examination often requires lengthy sample preparation. Earliest measurements of CBF utilized complex cinematography cameras coupled with high power microscopy to record single cilium throughout the entire beat cycle^7–9^. CBF was then calculated by analyzing the recording in slow motion and counting cilia cycles. The development of high speed digital cameras has replaced cinematography methods as the modern gold standard for CBF measurements as they are simpler to use and allow for faster analysis immediately after recording or even in real-time. While the recording mechanism has evolved, the measurement principles have remained the same and require visualizing and counting individual cilia beating cycles, albeit advancements have been made towards automating or semi-automating the process^4, 10, 11^. Other measurement methods have also been developed to analyze the frequency of light intensity fluctuations caused by ciliary motion using photomultiplier tubes^3, 12^ and photodiodes^13^. All of these approaches require the cilia samples to be studied under microscopy, either bright-field or phase contrast, and that the long axis of the cilia be oriented orthogonally to the illumination axis so that light isn’t blocked by the cilia substrate. Thus, none of these approaches are applicable for *in vivo* calculations of CBF in the respiratory tract where mucosal composition and environmental differences may alter the average beat frequency.

Optical coherence tomography (OCT) is an interferometric imaging technique capable of acquiring high-resolution micrometer scale cross-sectional images of scattering media^14^. OCT offers attractive features for the study of cilia in the upper airway, because it utilizes non-ionizing illumination that is safe for use in biological tissue and naturally performs optical sectioning which allows isolation of the cilia layer. Recently, a number of reports have utilized OCT to study ciliary function. Liu *et al*. used an ultrabroad supercontinuum laser to achieve 1 μm axial resolution OCT imaging which allows direction visualization of the cilia stroke cycle^15^. For most OCT systems, the size of individual cilia is smaller than the resolvable axial resolution. However, although not directly resolvable, motion of the cilia during the beating cycle still results in interactions with light that can be detected. Oldenburg *et al*. described a method to measure CBF in cultured human bronchial epithelial cells by correlating cilia induced speckle variation over time^16^. Wang *et al*. expanded this approach by performing the first *in vivo* measurements of CBF using OCT in the exposed oviduct of female mice^17^. OCT has also been used to study cilia driven fluid clearance as an analog to MCC by introducing polystyrene beads in a Xenopus model and tracking their speed and motion^18, 19^. While all of these approaches have their merits, none of them offer the ability to fully quantify cilia-based dynamics *in vivo*. Ultrabroad OCT requires expensive broadband waveguides and optics and also suffers from chromatic aberration effects, which makes it difficult to translate into *in situ* imaging in the upper airway. Speckle variance based methods can calculate CBF, however they cannot provide information about the synchronicity of beating cilia which is crucial for effective MCC. Fluid flow measurements require contrast agents, either dyes or particles, which limits their *in vivo* applications.

Doppler OCT (D-OCT) is a functional development of OCT that measures the motion or velocity of particles by detecting the Doppler induced phase shift of the back-scattered light^20–22^. The clinical utility of D-OCT has previously been reported with regards to detection of blood flow and tissue vibration^23–25^. As with all interferometry techniques, the phase sensitivity in OCT systems, typically in the picometer scale, is much higher than axial displacement sensitivities. In addition, relative phase changes can easily be quantified as displacement measurements since no complex phase wrapping occurs. During the cilia stroke cycle, upwards and downward motion of the cilium imparts a Doppler shift in the backscattered light, which results in a phase shift in the interferometric OCT signal. Detection and decoding of the Doppler signal allows for the quantitative measurement of the cilia power stroke and recovery stroke as well as measurement of CBF. Recently, Ansari *et al*. utilized a high resolution phase resolved spectral domain optical microscopy system to measure cilia dynamics in the mouse airway^26^. Compared to spectral domain systems, swept-source based OCT systems offer several attractive advantages including balanced detection and potentially higher signal to noise performance at higher speeds^27^. In the present study, we report on the use of swept source based Doppler OCT system to study sub-axial resolution cilia in *ex vivo* airway epithelial tissue samples and calculate ciliary dynamics such as height and beat frequency versus different environmental factors such as temperature as well as the presence of a therapeutic drug.

## Methods

### Cilia induced Doppler shift

The cilia beat cycle is broken up into two active parts: the effective or power stroke and the recovery stroke. The unidirectional transport of mucus or fluid is possible due to the asymmetric nature of the two strokes. During the power stroke, the cilia remain fully extended and swing through an arc like shape in a perpendicular plane to the tissue surface. A small portion of the cilia at the tip penetrates into the mucus layer generating forward propulsion^2, 28^. During the recover stroke, the cilia bend and swing backwards near the cell surface to reduce vertical height and prevent interference with forward fluid transport. This vertical change in ciliary height directly generates phase differences which are detected by D-OCT processing. Backscattered light from the cilia will have either a positive or negative phase shift corresponding to whether the cilia were undergoing an upwards or downwards trajectory. Therefore, ciliary motion and CBF can be directly calculated by detection of the phase changes in the OCT interferogram over time.

### OCT System Design

**Figure 1:**
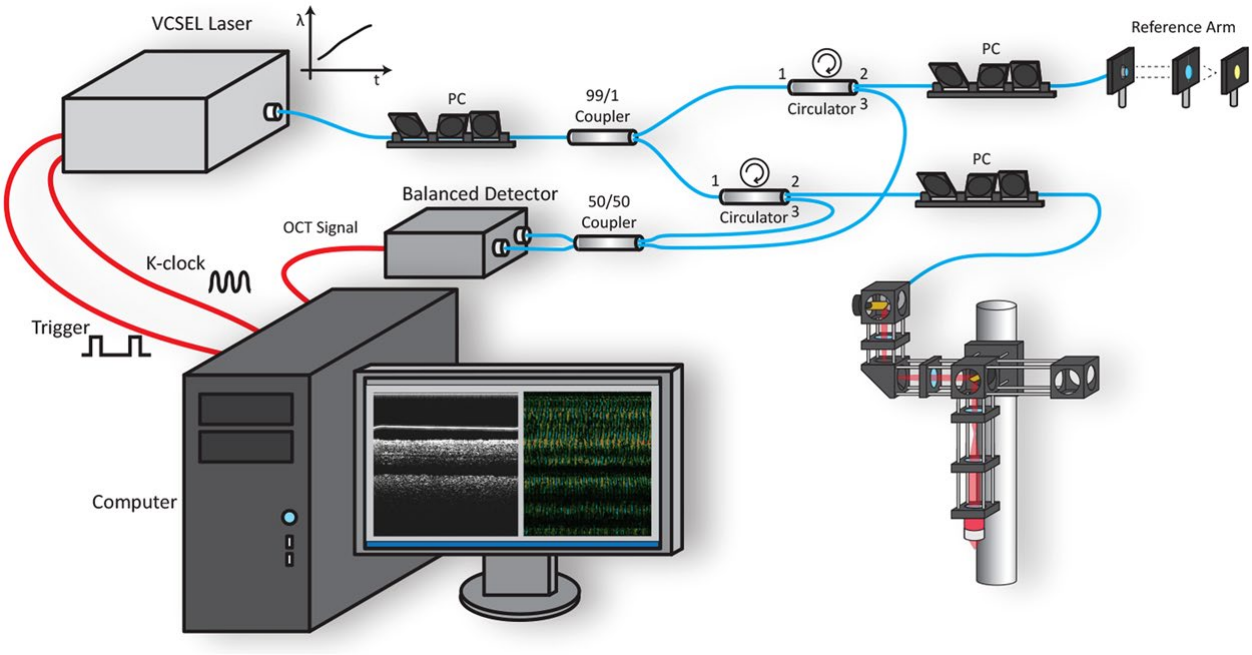
High resolution OCT system schematic.

As our method to measure CBF is based on the phase shift caused by ciliary motion, extra steps were taken to minimize all sources of phase noise within our OCT system. The OCT system utilizes a Mach-Zehnder based interferometer setup with a 99:1 fiber coupler, splitting the light between an optical delay line in the reference arm and a galvanometric scanner in the sample arm (Fig. 1). Dual circulators in both the reference and sample arms direct the back-scattered light to a 50:50 coupler at which point the resulting OCT interference fringe is detected utilizing a 1.6 GHz balanced photodetector. Anti-reflection coated optical windows were placed in the reference arm to compensate for extra dispersion in the sample arm. A commercial swept source vertical-cavity surface-emitting laser (VCSEL) was used as the light source for the system. The source features a center wavelength of 1310 nm, with a bandwidth of 100 nm, an average power of 26 mW, and a scan repetition frequency of 100 kHz. Compared to other swept source laser technologies, VCSEL sources feature a very short cavity length, which translates to both increased imaging range and phase stability allowing for improved performance in phase resolved techniques^29^. The OCT interferogram was captured using a high-speed 12-bit data acquisition (DAQ) card. Sampling was performed using the K-clock provided by the VCSEL so that the acquired data was linearly spaced in wavenumber, which is a crucial step for generating undistorted Fourier domain OCT (FD-OCT) images. A fiber Bragg grating, which generated a pulse every time the sweep of the VCSEL passed over a specific wavelength, was used to start each OCT A-line acquisition. This optical based trigger does not suffer from timing jitters and improves phase stability by ensuring that the starting wavelength is the same for each OCT A-scan.

The scanner in the sample arm features a design used in many laser scanning confocal microscopes^30, 31^. Two-dimensional scanning is achieved using two galvanometers and two telecentric relays. The first relay (f = 60 mm, magnification = 1) ensures that the sample light is centered along the rotational pivot axis for both mirrors to minimize phase shift artifacts from off axis scanning^32, 33^. The second relay (f_1_ = 35 mm, f_2_ = 75 mm) translates the pivot of the last galvo to the back focal plane of a long working distance objective (20x magnification, 10 mm working distance, 0.4 NA) to create a flat scanning plane across the cilia surface. A wavelength division multiplexer (WDM) was at the entrance to the scanner setup to allow incorporation of a red aiming beam to aid in visualizing the location of the scanning plane during imaging. The entire galvo scanner tower was mounted to a linear stage which was then attached to a damped optical post to axially tune the focal plane onto the ciliary layer of the samples. The entire OCT system, including the reference arm, sample scanner, and interferometry unit, was built on top of an anti-vibration optical table which was actively floating during imaging.

The system axial resolution depends only upon the bandwidth of the laser and was measured to be 8 μm by analyzing the full width half max (FWHM) of the point spread function (PSF) obtained from a mirror in the sample arm. Lateral resolution is a function of the focusing objective and was measured to be 1.2 μm by calculating the line spread function from the derivative of an edge response^34^. The phase stability of the system, defined as the standard deviation of the phase differences between sequential A-scans measured from the surface of a mirror in the sample arm^35^, was measured to be 2.9 mrad which translates to a displacement sensitivity of 0.3 nm in air.

### Doppler OCT

The principles behind D-OCT have been described previously^24, 27, 36^. Briefly, D-OCT processing involves extraction and measurement of relative phase differences from OCT A-lines to calculate motion or vibration within a scattering media. The phase difference can be empirically calculated using the cross-correlation method:

1 $${\rm{\Delta }}\varphi =[{\tan }^{-1}\{\frac{{\sum }_{j=1}^{J}{\sum }_{z=1}^{N}[{\rm{Im}}({A}_{j+1,z}){\rm{Re}}({A}_{j,z})-{\rm{Im}}({A}_{j,z}){\rm{Re}}({A}_{j+1,z})]}{{\sum }_{j=1}^{J}{\sum }_{z=1}^{N}[{\rm{Re}}({A}_{j+1,z}){\rm{Re}}({A}_{j,z})+{\rm{Im}}({A}_{j+1,z}){\rm{Im}}({A}_{j,z})]}\}]$$

where $J$ is the number of A-lines to average, $N$ is the number of depth points to average, and ${A}_{j,z}$ is the complex data of the ${j}^{{th}}$ A-line at a depth of $z$ after a standard Fourier transform utilized in FD-OCT setups. Generally, the A-scans in equation $(1)$ are separated by a time interval ${\rm{\Delta }}T$. Selection of a suitable time interval is an important consideration in D-OCT; too large of an interval can result in relative phase differences larger than $2\pi$ causing indistinguishable phase wrapping, while too small of an interval can hide lower frequency phase changes. For D-OCT based detection of ciliary motion, the axial changes in cilia height, ${\rm{\Delta }}z$, can be calculated using the relationship

2 $$\frac{{\rm{\Delta }}\varphi }{2\pi }=\frac{2n\cdot {\rm{\Delta }}z}{{{\rm{\lambda }}}_{0}}\,\Rightarrow {\rm{\Delta }}z=\frac{{\Delta }\varphi \cdot {\lambda }_{0}}{4\pi \cdot n}$$

where ${\lambda }_{0}$ is the center wavelength of the OCT light source, $n$ is the index of refraction of the mucosa layer, and the extra factor of 2 represents the double pass distance of the sample light. Through equation $(2)$ the Doppler induced phase shift can be translated into a more meaningful measurement of height changes that occur throughout the entire cilia beating cycle and determination of CBF.

### ***Ex vivo*Tissue Sample Preparation**

*Ex vivo* samples from the nasal septum, trachea, and maxillary sinus were harvested from freshly euthanized 3.4–4.2 kg male New Zealand white rabbits under the approval and in accordance with the regulations of the Institutional Animal Care and Use Committee (IACUC) at UC Irvine under protocol 2004–2553. The samples were submerged in buffer solution (Hanks Balanced Salt Solution, HBSS) and maintained at room temperature (21–23 °C) while microdissection techniques were used to remove excess soft tissue. Then, 1–2 mm thick samples were sectioned from the samples and mounted with pins on rubber lined culture dishes with the mucosal surface facing upwards. The samples were submerged so that a thin layer of buffer covered the surface of the mucosa and were transferred to the scanner stage for imaging. For a control, samples were fixed in 10% formalin for 30 minutes and imaged again with D-OCT (Supplemental Fig. 1).

### Imaging Setup and Processing for Calculating CBF

Cilia samples were positioned under the OCT scanner for imaging with the aid of a red aiming beam. The height of the scanner was adjusted using a linear stage such that the cilia layer, identifiable as a layer of fluctuating speckle, was brought into focus which was facilitated by the objectives short depth of focus. OCT B-scan imaging was then performed by continuously acquiring data over a period of 10 seconds while repeatedly line scanning across a section of the tissue sample. The acquired OCT data was then resampled to obtain a series of M-mode data sets at different points along the scanned line. Doppler OCT processing was performed on each M-mode scan to extract the temporal phase variations caused by ciliary motion. Control of the time interval between adjacent A-scan acquisitions in the M-mode data sets for optimized Doppler processing was achieved by changing the imaging frame rate of the original scan, i.e. the B-scan density.

**Figure 2:**
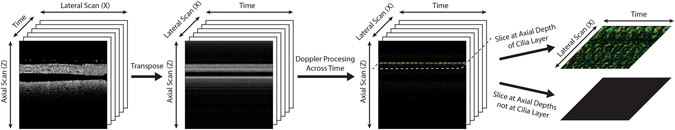
Processing flowchart for calculating CBF using Doppler OCT. The M-mode slice at the cilia layer will show the induced phase shift from ciliary motion while all other layers will have very low phase signals. The axial scan direction (Z) refers to the propagation direction of the scanning light into the cilia sample while the lateral scan (X) is parallel to the surface of the cilia.

All data processing was performed using commercial graphics processing units (GPU) which allowed for massive parallelization of the D-OCT algorithms. A flowchart of the data processing steps is shown in Fig. 2. First the acquired series of B-scans is loaded into memory and then resampled to generate a series of spatially adjacent M-mode data sets. These data sets are transferred into GPU memory where first numerical dispersion compensation is applied to each A-line followed by a Fourier transform to generate the complex OCT scattering profile as a function of depth. The OCT intensity image is calculated by taking the magnitude of the complex axial profile. Meanwhile, a Doppler color image, where red represents $+\pi$ phase shift and blue represents $-\pi$ phase shift, is generated by calculating the relative phase changes between adjacent A-lines in a frame. The OCT intensity image is used as a mask to obscure the random phase signals that don’t originate from backscattering tissue. M-mode slices at different depths are generated from the Doppler images to better visualize the temporal ciliary dynamics. Identification of the cilia layer is greatly simplified since at all other axial depths the sample is largely static which generates a very small Doppler signal. The Doppler phase signal can finally either be converted and plotted to show the changes in cilia height over time or processed using a Fourier transform to show the CBF frequency distribution.

## Results

### *Ex vivo* cilia imaging

**Figure 3:**
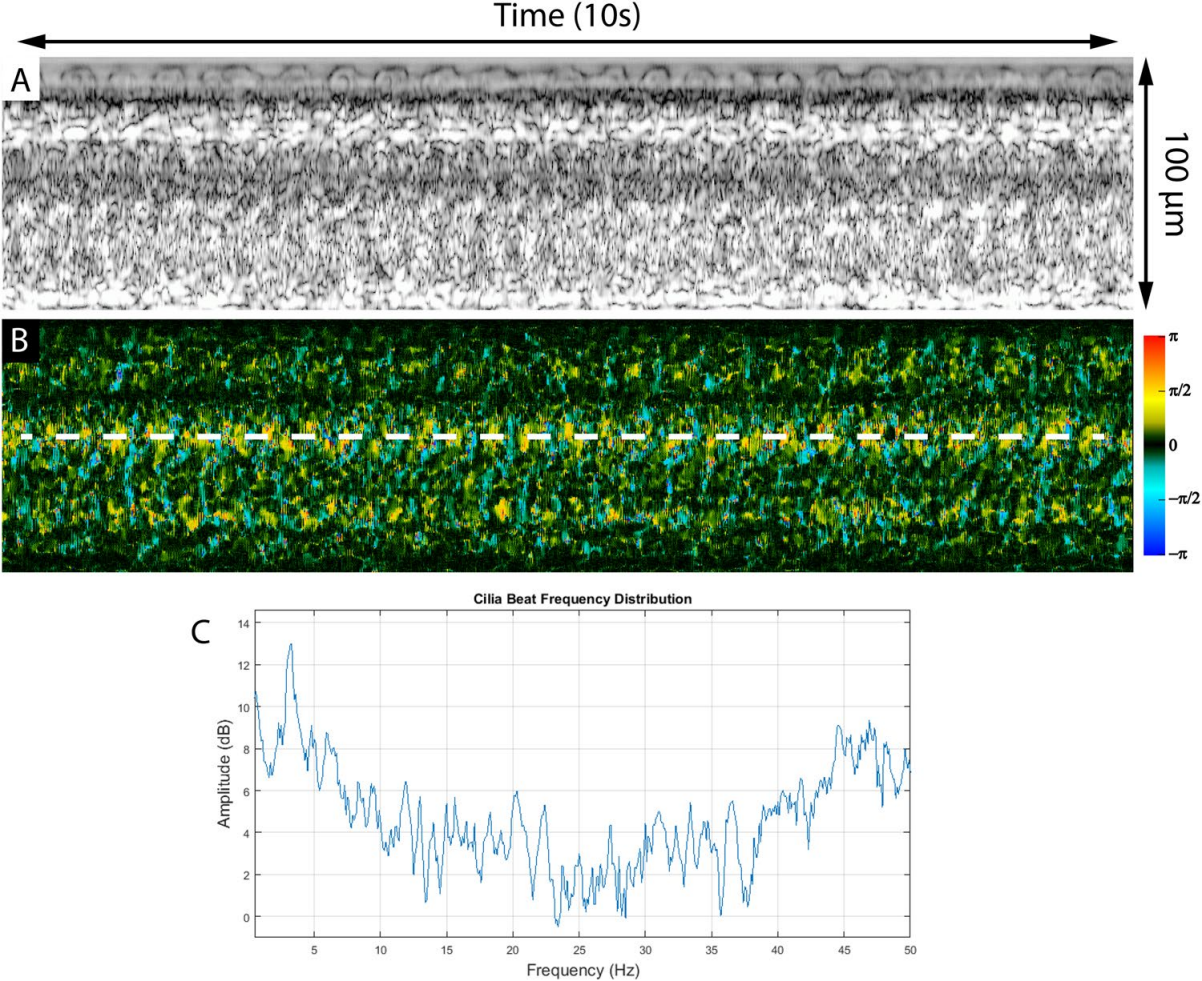
M-mode intensity image at the cilia layer scanned over time (**A**). Doppler M-mode image over time showing ciliary induced phase shift (**B**). Fourier transform of phase shift in (**B**) showing a frequency of 3.8 Hz (**C**).

Imaging was first performed on *ex vivo* trachea samples acquired as stated in the methods section. OCT data was acquired and Doppler processing was computed using equation (1) with an averaging of 2 across adjacent A-scans and no averaging across depth. M-mode slices at each axial depth were generated from the OCT intensity and Doppler images to isolate the thin cilia layer. Figure 3 shows a M-mode slice acquired from imaging a tracheal sample. Imaging was performed at a B-scan rate of 100 Hz which set the interframe time interval to be 10 ms. Comparison between the M-mode intensity slice and Doppler phase slice reveals a clear difference in ability to distinguish ciliary motion. In the intensity image, a faint periodic change in signal intensity caused by speckle variation from moving cilia is faintly visible through the middle of the image. Meanwhile, in the Doppler image, the periodic ciliary motion is much more easily visualized and separate portions of the beat cycle can also be distinguished. During the power stroke, the cilia have a rapid downward motion identified by the shorter blue segments while during the recovery stroke the cilia have a much more gradual upwards trajectory characterized by the longer yellow/red sections. A Fourier transform of the phase variation signal yields the CBF which for this sample was measured to be 3.8 Hz. Previous studies have reported rabbit tracheal CBF to be approximately 8.3 Hz but can vary anywhere between 7.5–22.4 Hz^37, 38^. The low CBF can possibly be explained by the fact that our tissue samples were kept in room temperature buffer instead of at 37 °C since CBF is well known to have a dependence on temperature^39–41^. Confirmation of the CBF was done using visual inspection of high-speed digital video captured using a separate microscope (32x, 0.4 NA)^42^. As a control, after imaging samples were exposed to a 10% formalin solution and the absence of any ciliary motion, both in intensity images and phase images, was verified.

### Imaging of ciliary motion vs temperature

To study the effects of temperature on CBF, we performed D-OCT imaging on tissue samples at various temperatures. Samples were taken from *ex vivo* rabbit nasal septa and submerged in room temperature in preparation for imaging. A heating plate with temperature feedback was mounted on top of a two-axis linear stage positioned underneath the sample scanner. A plastic petri dish (100 mm diameter, 15 mm depth) was placed on top of the heating plate and filled with HBSS. The samples were then positioned into the petri dish such that the tissue surface was just fully submerged by the buffer. The excess buffer within the petri dish acted as a temperature buffer allowing for finer gradual increases in temperature from the heating plate. OCT imaging was performed at buffer solution temperatures of 25 °C, 27 °C, 29 °C, 31 °C, and 34 °C after allowing the submerged cilia five minutes to reach equilibrium. Temperature monitoring was achieved using three thermometers: the feedback probe included with the heating plate, a digital infrared thermometer, and a spirit filled glass thermometer.

**Figure 4:**
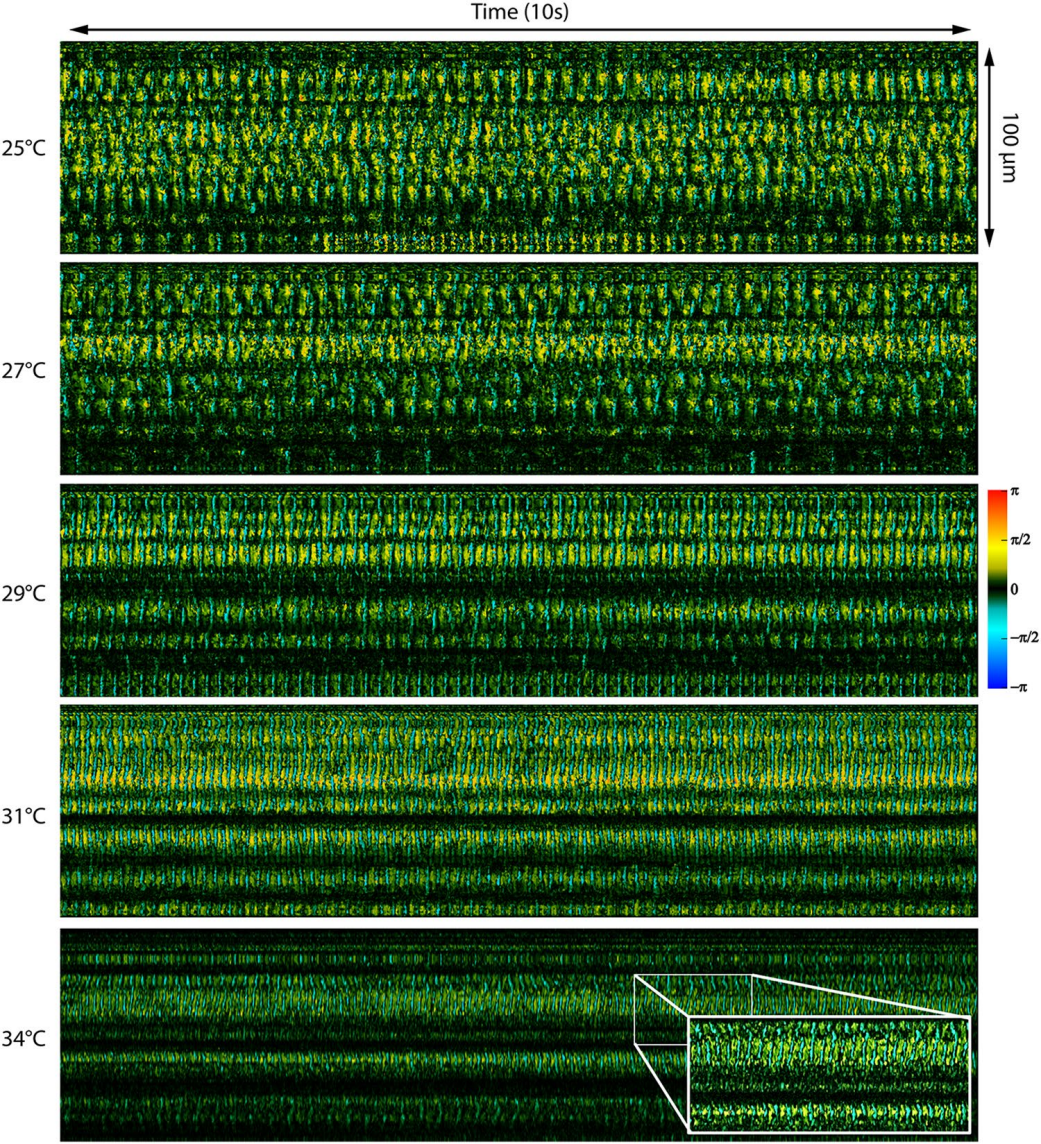
Doppler images of ciliary motion over time. Acquired at temperatures of 25 °C, 27 °C, 29 °C, 31 °C, and 34 °C. 10 seconds duration. Red/yellow represents periods of upward motion while blue/turquoise represents periods of downward motion.

**Figure 5:**
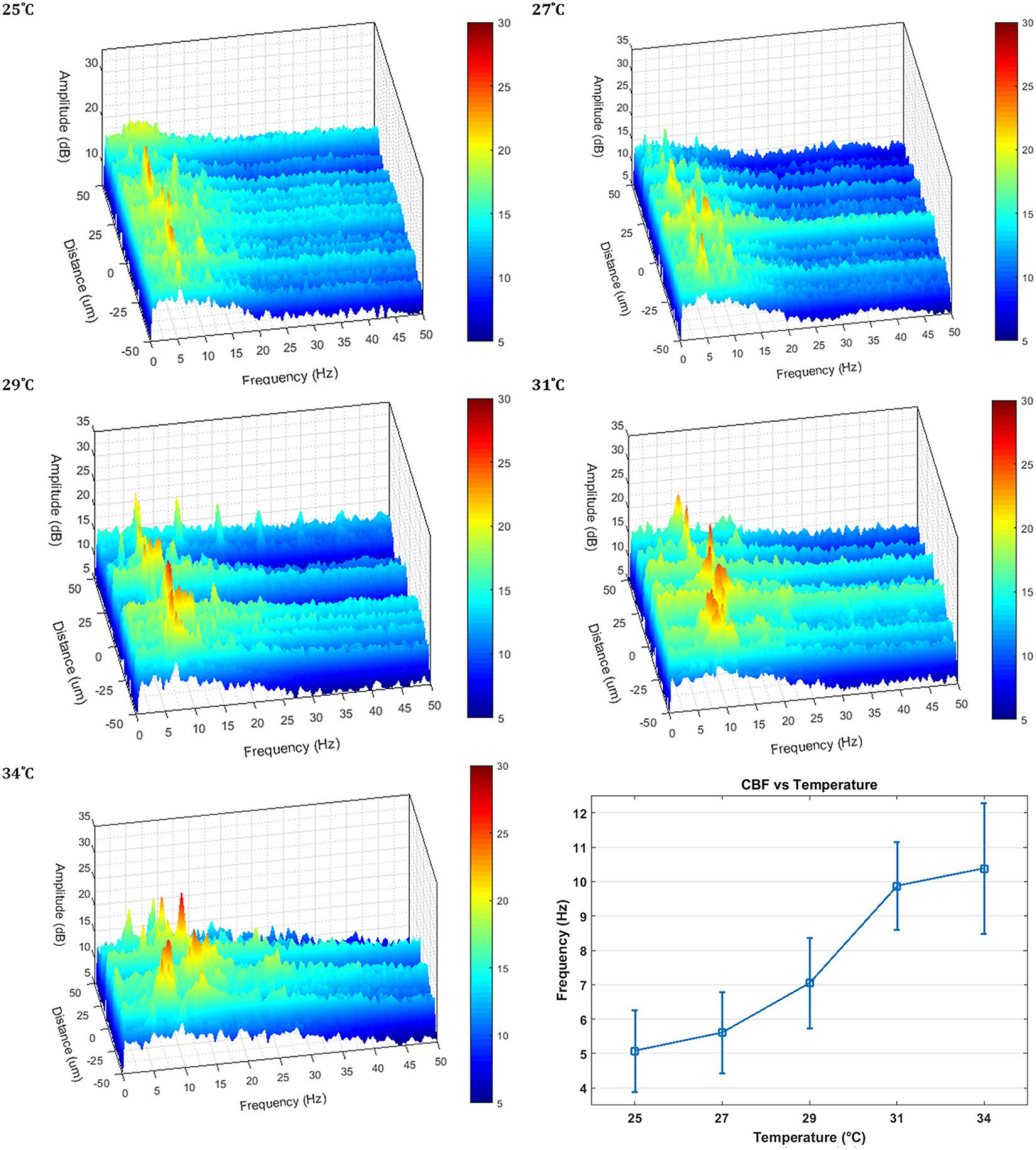
Power spectral density graphs showing peak CBF as a function of space across the sample at temperatures of 25 °C, 27 °C, 29 °C, 31 °C, and 34 °C. Graph of average CBF as a function of temperature.

**Figure 6:**
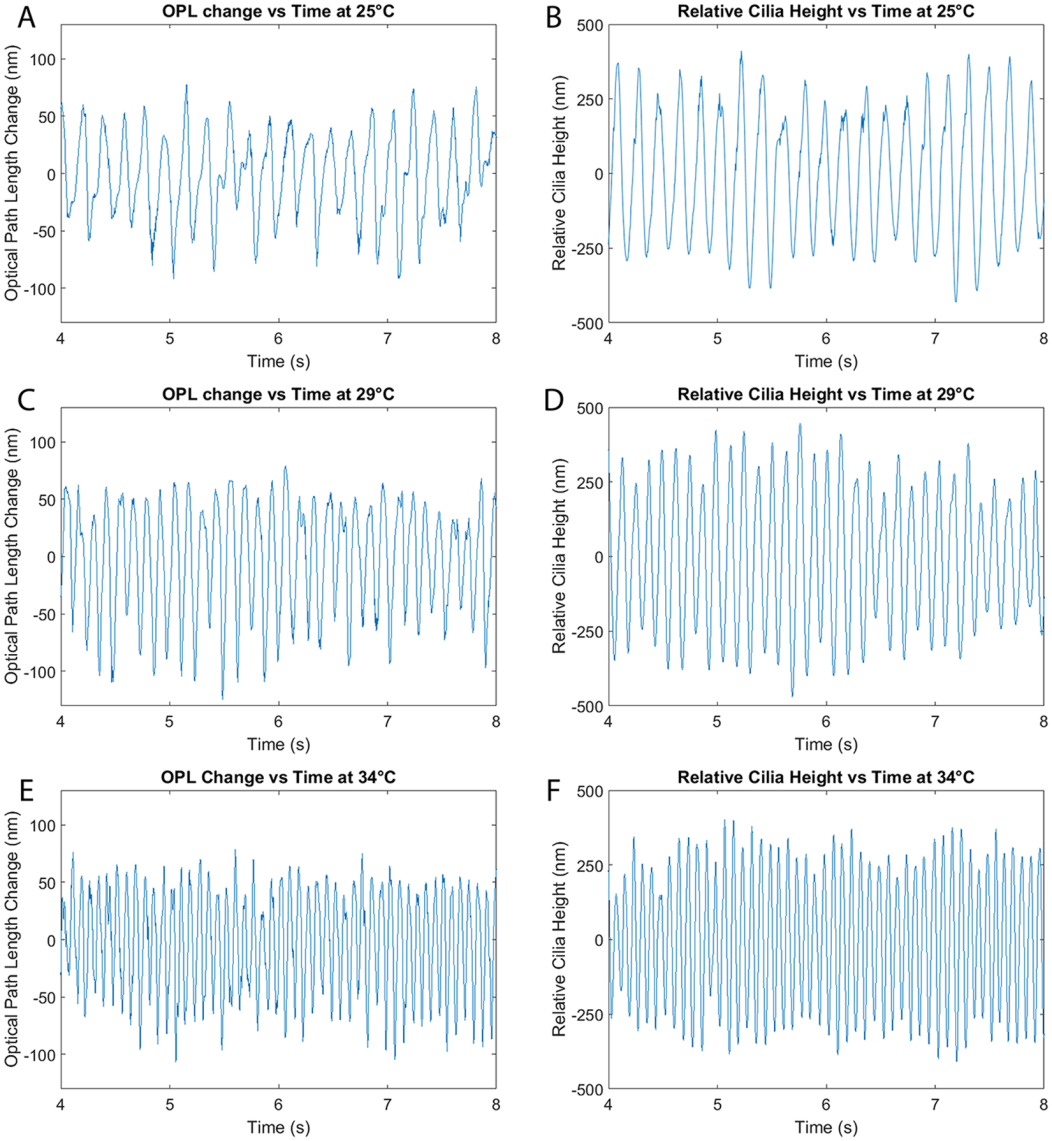
Axial displacement of cilia between sequential time points at 25 °C, 29 °C, and 34 °C (**A**,**C**,**E**). Time integration of axial displacement showing temporal measurements of cilia height at 25 °C, 29 °C, and 34 °C (**B**,**D**,**F**).

Figure 4 shows M-mode slices of Doppler phase images from a nasal septum sample scanning the same location at 25 °C, 27 °C, 29 °C, 31 °C, and 34 °C. Special care was taken to align the scanning axis orientation to be perpendicular to the ciliary wave propagation direction to maximize spatial cilia visibility. Figure 5 shows three-dimensional power spectral density (PSD) plots of the ciliary frequency distribution across the scanned area. Imaging was continuously acquired over a 10 second period for each acquisition with the frame rate tuned at each temperature point to prevent phase wrapping. B-scan frame rates corresponding to the temperature points (25 °C, 27 °C, 29 °C, 31 °C, and 34 °C) were set at 200, 200, 250, 250, and 400 Hz respectively. A mean CBF was calculated for each temperature point by averaging the peak frequencies across the scanning range. CBF was measured to increase from 5.1 Hz at 25 °C to 10.4 Hz at 34°C. The gradient of the CBF temperature relationship also was in agreement with previous reports of cilia temperature effects where a sharper slope is seen when the temperatures are in the range of 25–32 °C but plateaus in the more physiological temperature range of 32–40 °C^39^. To quantify changes in cilia height, phase shift values were extracted from the Doppler images and converted to displacement measurements using equation (2) with an index of refraction of n = 1.33^43^. Figure 6 shows the relative changes in cilia height between sequential time points (a,c,e) as well as the time integrated change in cilia height (b,d,f) at the same spatial location and three temperature points and verifies that our system is able to detect axial displacements much smaller than our system’s axial resolution. Higher temperatures were not imaged for this sample as the spatial density of our B-scans was already becoming under-sampled at 34 °C leading to phase wrapping. However, future imaging can be performed at high temperatures by using a faster laser to avoid spatial under-sampling.

### Imaging of ciliary motion vs drug application

**Figure 7:**
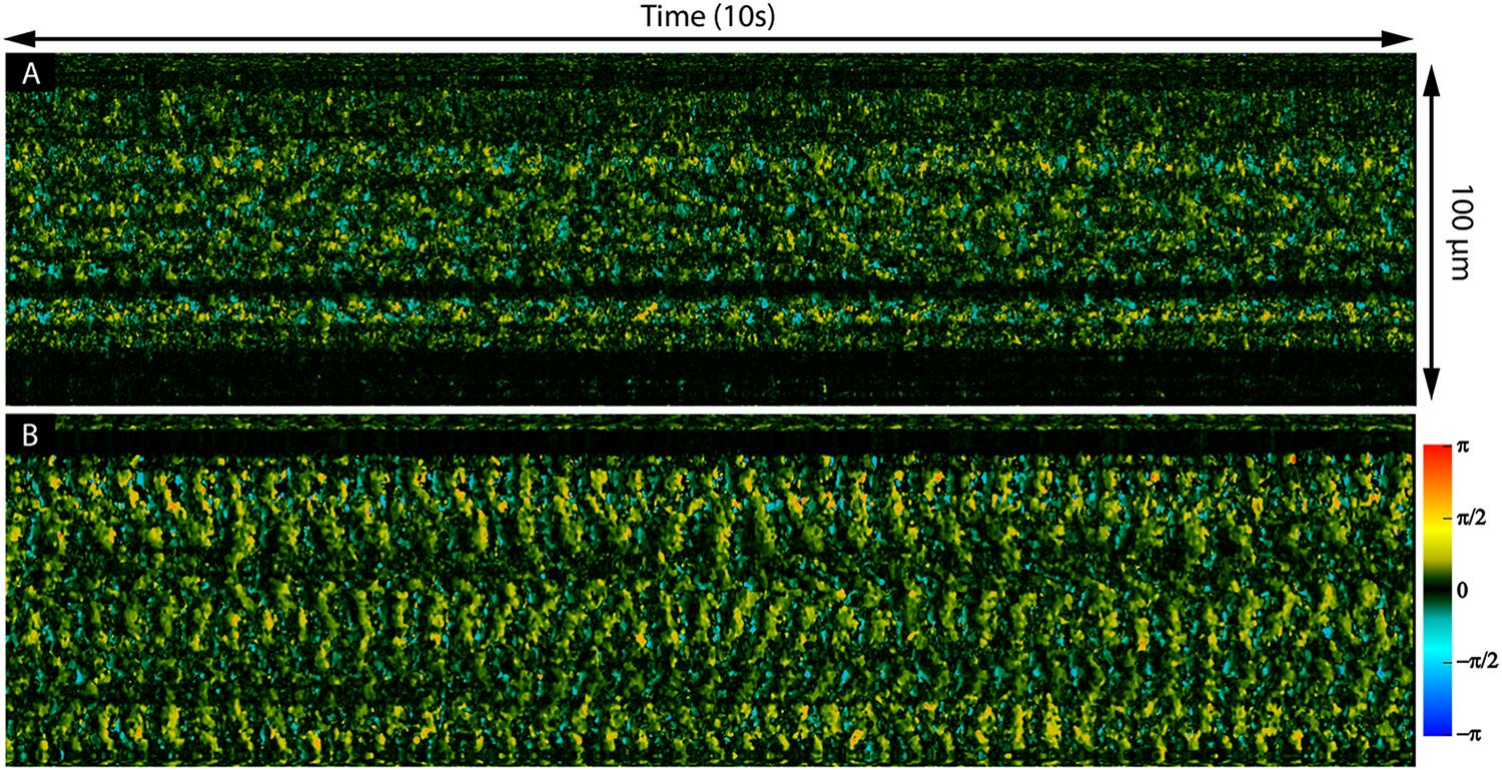
Phase resolved ciliary motion in rabbit nasal mucosa without treatment (**A**) and with albuterol treatment (**B**). All acquisitions were scanned over a 100 um line for 10 seconds.

Several reports have linked increased CBF and MCC with the application of therapeutic drug agents for improving airway respiratory function^44–46^. We performed a preliminary investigation to see if our D-OCT system could visualize increased CBF in the presence of albuterol, a therapeutic agent used to treat COPD and shown to improve MCC^44, 45^. D-OCT imaging was performed on *ex vivo* nasal mucosa from the maxillary sinus of a rabbit. The sample was submerged in room temperature buffer (23 °C) to keep phase signals generated by the beating cilia from wrapping. A solution containing 2 mg of albuterol diluted in 30 mL of buffer at room temperature was prepared and topically applied to another sample cut from the same tissue section and imaged with the D-OCT system. Figure 7 shows the corresponding temporal phase variation image for the untreated (Fig. 7A) and treated samples (Fig. 7B). The measured CBF increased from 3.18 Hz in the untreated sample to 4.6 Hz for the albuterol treated sample with standard deviations of 0.89 Hz and 0.49 Hz respectively.

## Discussion

Cilia play a crucial role in protecting the airway from pathogens and irritants, and CBF has been demonstrated to be an important physiologic measure of overall respiratory mucosal health. The measurement of CBF has the potential to provide clinicians with a new tool to study various cilia related upper airway diseases, such as CF and PCD, as well as quantify the efficacy of different airway therapies. The current gold standard for measuring CBF is direct visualization using high-powered microscopy combined with digital high-speed cameras. Imaging is limited to studying harvested cilia samples in tissue culture which may have an altered native CBF. While advances have been made towards automated detection of CBF, the vast majority of measurements are made by visually counting ciliary beat cycles; a process that is extremely time consuming and tedious and somewhat subjective. Likewise, the synchronicity of the cilia beating pattern which plays a crucial role in overall cilia efficiency may also be affected by the cilia no longer being in their native environment. Thus, microscopy based approaches will likely remain unsuitable as a rapid diagnostic tool of ciliary behavior in a clinical setting.

Doppler OCT imaging offers many attractive features for the study of cilia. D-OCT generates cross-sectional images of tissue layers in which motile regions of interest can very quickly be identified and brought into focus. Since D-OCT has inherent axial sectioning and does not rely on resolving individual cilium, objectives with more moderate numerical apertures that do not require water or oil immersion can be used for focusing. Tissue samples also do not require special handling or orientation for imaging unlike conventional microscopy approaches. Ciliary motion can be detected based on vertical changes in cilia height and unlike in microscopy methods, this change height can be measured throughout the beating cycle. In comparison to other OCT imaging approaches, D-OCT also offers several attractive benefits. In previously reported ultra-high resolution OCT approaches, the differences in cilium height during the beat cycle were on par with the axial resolution of the imaging system which made identifying where the cilium were in the beating cycle difficult. In contrast, with D-OCT the power (blue) and recover stroke (yellow) as well as the asymmetric time period of each stroke are clearly identifiable by their differing phase shifts. D-OCT also has the capability to rapidly visualize the temporal beating synchronicity of adjacent cilia across space, which other correlation based OCT methods lack and is as important a feature as CBF for effective mucociliary clearance. Various studies have also demonstrated the translation and compatibility of D-OCT based measurements in the upper airway^47^. While our current approach relies on a large external sample arm setup, the scanning mechanism can be reduced and packaged into a form that is conducive for *in vivo* imaging^47^. Several reports have demonstrated the ability of using compact gradient index (GRIN) lenses to achieve ultrahigh lateral resolution imaging with numerical apertures of up to 0.65–0.85^48–50^ which is suitable for performing D-OCT cilia imaging.

Doppler OCT does require extra measures to maximize system sensitivity for the study of cilia. First, phase stability is of paramount importance and steps must be taken to minimize all sources of phase noise in the system. Recent advances in laser designs have made swept source based OCT phase stability nearly on par with spectral domain systems, and various reports have demonstrated the capability of removing noise that can arise from clock glitching or trigger jitter. Second, while a system with high axial resolution is not critical, high lateral resolution is required for proper cilia detection. If the lateral resolution is too large, phase from additional cilia at different points in the beating cycle will sum together and wash out any signal. Third, in order to prevent phase wrapping in the Doppler data, OCT imaging has to be performed at much faster rates than the Nyquist frequency. Many of our samples were imaged in room temperature buffer rather than the standard physiological temperature of 37°C in order to reduce CBF to prevent phase wrapping artifacts; however, measured CBF nearer to physiological temperatures agreed with measured values in literature^39^. While currently limited by laser scanning speeds, the continued introduction of newer and higher speed lasers will allow for visualization of faster CBFs at true physiological temperatures. Higher speed lasers that operate in megahertz range also open the possibility of performing D-OCT imaging across two-dimensional space to better visualize wide-field cilia dynamics *in situ*. Full field OCT also has potential for wide-field cilia imaging since an entire *en face* plane is capture simultaneously. However, a potential drawback lies its reduced SNR which cannot be aided by techniques such as averaging since the cilia motion is dynamic. Recent works on full field swept source OCT systems demonstrate the ability to combine together full field and Fourier domain OCT^51, 52^. These systems utilize ultrahigh speed area cameras together with swept source based OCT illumination to allow for scanner-less based wide-field imaging while still offering the SNR performance of Fourier domain OCT and thus excellent potential for high speed volumetric phase imaging of cilia dynamics.

Doppler based swept-source OCT allows for rapid and objective visualization of cilia dynamics that play crucial roles in mucociliary clearance. The period of the cilia cycle can be clearly identified based on the induced phase shift caused by the changes in cilia height through the power and recovery strokes. CBF was shown to increase with temperature and measured CBF values were shown to agree with values published in literature. In addition, cilia beat frequency also demonstrated a marked increase with the application of Albuterol showing the potential of using D-OCT for *in situ* studies of ciliary dynamics. Our current approach lays the framework for our ongoing development of an *in vivo* cilia imaging platform which can provide important information in the study of various cilia related airway disorders.

## Electronic supplementary material

Supplemental Figure
