# Supplementary material for: Visualization and Detection of Ciliary Beating Pattern and Frequency in the Upper Airway using Phase Resolved Doppler Optical Coherence Tomography
Source: Sci Rep. 2017 Aug 17;7:8522. doi: 10.1038/s41598-017-08968-x (PMC5561030; doi:10.1038/s41598-017-08968-x)
Supplement: Supplementary file 1 — Supplemental Figure [file 41598_2017_8968_MOESM1_ESM.pdf]

Visualization and Detection of Ciliary Beating Pattern and Frequency in the Upper Airway using Phase Resolved Doppler Optical Coherence Tomography

Joseph C. Jing[1,2], Jason J. Chen[1], Lidek Chou[1], Brian J.F. Wong[1,2,3], Zhongping Chen[1,2,*]

[1]Beckman Laser Institute, University of California Irvine
[2]Department of Biomedical Engineering, University of California Irvine
[3]Department of Otolaryngology—Head and Neck Surgery, University of California Irvine

*Corresponding author:

Zhongping Chen, PhD
Professor
Dept. of Biomedical Engineering
University of California, Irvine
Irvine, CA 92617
e-mail: z2chen@uci.edu

Supplemental Figures

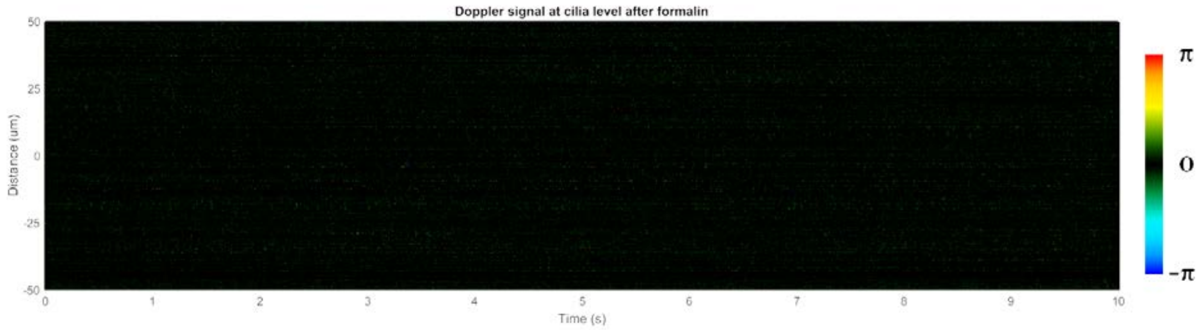

Supplemental Figure 1. Background Doppler signal from cilia sample after exposure to 10% formalin solution
